# GluD1 at the synaptic crossroads: from domain structure to circuit dysfunction

**DOI:** 10.1038/s41401-025-01696-3

**Published:** 2025-12-04

**Authors:** Poojashree B. Chettiar, Shashank M. Dravid

**Affiliations:** Department of Psychiatry and Behavioral Sciences, Naresh K. Vashisht College of Medicine, Texas A&M University, College Station, TX USA

**Keywords:** inotropic glutamate receptors (iGluRs), glutamate delta-1 receptor (GluD1), synaptic plasticity and organization, trans-synaptic signaling, modular receptor domains

## Abstract

For several decades, the glutamate delta-1 receptor (GluD1) has remained an enigmatic entity among ionotropic glutamate receptors (iGluRs), primarily due to its lack of classical ion channel activity. Recent advancements have redefined GluD1 as a multifunctional synaptic organizer, essential for the development, plasticity, and behavioral regulation of both excitatory and inhibitory circuits. In this review, we synthesize recent progress at the structural, molecular, and circuit levels to reconceptualize GluD1 as a pivotal signaling scaffold that functions through non-ionotropic mechanisms. We emphasize the modular architecture of GluD1, encompassing the amino-terminal domain, ligand-binding domain, transmembrane region, and C-terminal domain to elucidate how each component uniquely contributes to synaptic function. Evidence from genetic models and structural biology underscores GluD1’s involvement in transsynaptic adhesion, ligand-dependent conformational signaling, and intracellular pathway modulation. Additionally, we discuss its emerging clinical significance, with GRID1 mutations associated with neurodevelopmental and psychiatric disorders, and recent findings implicating GluD1 dysfunction in chronic pain. Finally, we explore domain-specific therapeutic strategies, including peptide mimetics, synthetic organizers, and non-ionotropic modulators, positioning GluD1 as a promising target for circuit-level intervention in brain disorders.

## Introduction

Glutamate delta 1 and delta 2 receptors (GluD1 and GluD2) make up the enigmatic delta subfamily (GluDs) of ionotropic glutamate receptors (iGluRs). While they share a modular architecture comprising of an extracellular amino-terminal domain (ATD), ligand-binding domain (LBD), transmembrane ion pore-forming region (TMD), and cytoplasmic C-terminal domain (CTD) with α-amino-3-hydroxy-5-methyl-4-isoxazolepropionate (AMPA), kainate, and *N*-methyl-*D*-aspartate (NMDA) receptors, GluDs lack a well-defined pore-opening ligand [1–4]. Their function as canonical ion channels has been called into question as a result. Nonetheless, their significance in synaptic organization and plasticity is becoming evident. GluD2 is known for its role in motor coordination, cerebellar synapse development, and plasticity [1, 5–8]. GluD1 shares 56% sequence similarity with GluD2 and is expressed across the forebrain, including the cortex, striatum, hippocampus, and amygdala [5, 6, 9], where it governs processes like regulating spine development [10], autophagy signaling [11], behavioral flexibility [12] and nocifensive and affective components of chronic and neuropathic pain [13].

This functional plasticity is reflected in its clinical significance. Genome-wide association studies have identified variants in the GRID1 gene, which encodes GluD1, as a risk factor for neuropsychiatric disorders like autism, developmental delay, bipolar disorder, and schizophrenia [14–19]. Consistent with this, GluD1 knockout mouse (GluD1 KO) models exhibit key behavioral endophenotypes linked to these conditions, such as social impairments, hyperactivity, and reduced cognitive flexibility [9, 20, 21]. Additionally, our recent research has revealed GluD1’s involvement in chronic pain processing, particularly in the central amygdala, which regulates affective responses to painful stimuli [13, 22–25].

In this review, we summarize developments that reframe GluD1 as a multifunctional synaptic organizer that integrates structural and signaling elements across excitatory and inhibitory networks, influencing circuit function in both healthy and pathological conditions. We emphasize the emerging therapeutic relevance of this glutamate receptor, highlighting recent insights into GluD1’s roles in synaptic plasticity, neuromodulation, and pain circuit remodeling.

## GluD1 expression and localization to unique synapses and relevance to behavior

Mapping GluD1 distribution was a major turning point in studying this receptor, providing crucial information to probe its function in brain circuits. Konno et al. and Hepp et al. first showed large-scale expression of GluD1 in the forebrain. These studies also demonstrated GluD2 expression in the forebrain, correcting earlier perceptions of selective GluD2 expression in the cerebellum [5, 6, 9]. Enriched GluD1 expression was observed in dorsal striatum, nucleus accumbens (NAc), lateral habenula, bed nucleus of the stria terminalis (BNST), lateral septum, lateral nucleus of central amygdala, and cerebellar interneurons. Within cortical and hippocampal structures, its expression shows layer-specific and projection-specific distribution. In the cortex it is enriched in layers 2/3 and 4 presumably postsynaptic to thalamic projections. In the hippocampus, GluD1 is expressed postsynaptically in lacunosum moleculare at projections from entorhinal cortex which are enriched in Cbln1 and Cbln4, in CA2 presumably postsynaptic to supramammillary nucleus projections and also in the dentate gyrus region postsynaptic to entorhinal projections [5, 6, 9].

This expression pattern is widespread yet anatomically distinct. GluD1 is found at axo-somatic synapses in cerebellar interneurons, the central amygdala and BNST potentially important to regulate excitability [13, 26]. Furthermore, ultrastructural localization of GluD1 in mouse and monkey striatum, reported enriched expression at dendritic shafts of striatal spiny neurons and NG2-positive oligodendrocyte progenitor cells (OPCs) [27, 28]. In the lateral habenula, GluD1 is enriched at symmetric GABAergic synapses formed by projections from the lateral hypothalamus and entopeduncular nucleus [29].

Beyond its localization, GluD1 has an active role in excitatory and inhibitory synapse development [30–32]. While GluD2 and Cbln1 were initially known as essential for parallel fiber–Purkinje cell (PF-PC) synapse formation [33, 34], early functional studies in GluD1 KO showed no significant changes in input-output ratio at CA3-CA1 synapses and no change in LTP [35]. Additionall, studies in GluD1 KO noted an increase in synapse number and increase in neurotransmission in the cortex which is contrary to its potential synaptogenic action suggesting potential compensatory effects [10]. Nonetheless, overexpression and knockdown of GluD1 leads to an increase and decrease in hippocampus and somatosensory cortex respectively [36]. Contrasting to this study, in the CA1 and subiculum loss of GluD1 or GluD1 knockdown does not affect excitatory neurotransmission [12, 37]. However, subsequent studies in GluD1 KO revealed that GluD1 loss produces regional and input-specific deficits in synaptic structure and function. In the dorsal striatum, GluD1 localizes to thalamostriatal terminals from the parafascicular nucleus, shown by its colocalization with vGluT2-positive puncta and axo-dendritic expression. Conditional deletion of GluD1 led to selective loss of thalamic excitatory inputs, reduced mEPSC frequency, and impaired behavioral flexibility without affecting corticostriatal vGluT1 input [12]. In the central amygdala, its deletion reduces AMPAR-mediated responses and excitatory terminal density, supporting its role as an essential postsynaptic organizer in non-cortical excitatory circuits [13]. Thus, although in vitro studies demonstrate that GluD1-Cbln signaling can induce synapse formation [30–32], the role in native system appears a bit complex. Overall, in the native system, the effect of GluD1 manipulation is dependent on specific circuits and likely relies on other synapse organizers which may compensate for the lack of GluD1. Consistent with the complex nature of this signaling, ablation of Cbln1 or Cbln2 leads on to an unusually delayed reduction in excitatory synapses in the hippocampus [38].

Initially recognized for its role in organizing excitatory synapses, GluD1 is now increasingly implicated in the regulation of inhibitory synapses. In cortical circuits, GluD1 contributes to the formation of inhibitory synapses from somatostatin-expressing interneurons through interactions with Cbln4, requiring structural integrity of its ATD, LBD, and CTD domains. Binding of ligands such as D-serine and glycine may be necessary for initiating downstream signaling through scaffold proteins such as PPP1R12A and ARHGEF12, supporting a non-ionotropic, scaffold-based mechanism of synapse modulation [39]. Disruption of GluD1 leads to diminished IPSC and reduced inhibitory synapse number and these effects persist even when channel conductance is abrogated. Notably, GABA binding to the GluD1 LBD is necessary for potentiation in response to high-frequency stimulus, without opening the ion channel pore, indicating a conformational signaling role dependent on trans-synaptic anchoring [40]. In the NAc core, GluD1 is localized to both excitatory and inhibitory synapses, confirmed by colocalization with excitatory markers (vGluT1 and vGluT2) and inhibitory marker GAD67 [41]. However, in GluD1 knockout mice, synaptic alterations were selectively observed at inhibitory synapses, with a significant reduction in GAD67-positive puncta and decreased amplitude and frequency of mIPSCs. Electron microscopy further confirmed GluD1’s distribution in dendritic spines and shafts, with signal detected at symmetric axo-dendritic synapses using gold-conjugated antibody tagging. These GABAergic synaptic deficits were accompanied by behavioral abnormalities when GluD1 was conditionally ablated from the NAc, including hypolocomotion and heightened anxiety-like behavior, underscoring GluD1’s essential role in maintaining excitation-inhibition balance within circuits mediating affective and motivational behaviors [41]. Consistent with the discussion above, the lack of changes in mEPSC in NAc upon GluD1 ablation and strong reduction in mIPSC suggest that GluD1 is obligatory for inhibitory but not for excitatory synapses in this region likely due to compensatory effect of other synapse organizers [41]. It will be important to examine whether the effect of GluD1 on inhibitory neurotransmission is more consistent, especially as it relates to somatostatin interneuron induced inhibitory synapses.

**Fig. 1:**
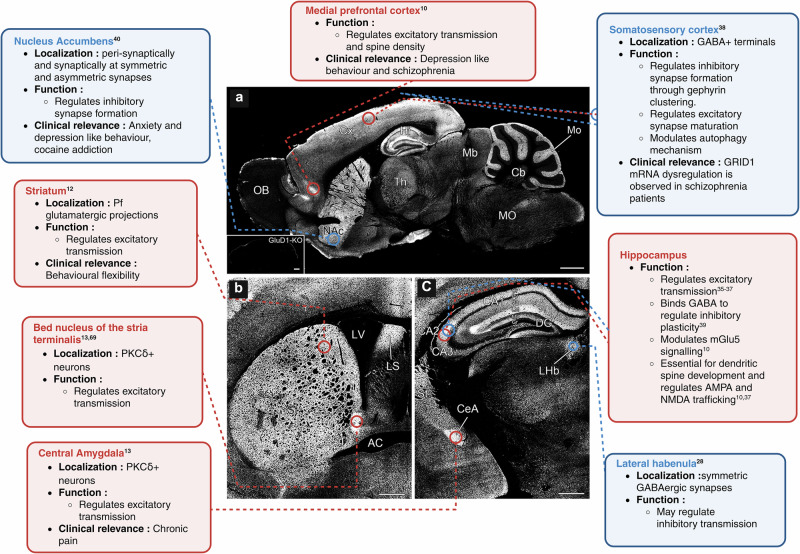
Region-specific localization and functional diversity of GluD1 in the rodent brain. Sagittal and coronal brain sections depict GluD1 expression across principal forebrain regions, with annotations regarding functional roles and clinical significance. **a** The sagittal section emphasizes GluD1 expression in the medial prefrontal cortex (Cx), striatum (St), nucleus accumbens (NAc), hippocampus (CA regions and DG), and lateral habenula (LHb). **b** The coronal section illustrates GluD1 localization in the bed nucleus of the stria terminalis (BNST) and anterior commissure (AC) regions. **c** A high-magnification sagittal section reveals GluD1 expression in the central amygdala (CeA), hippocampal subfields, and lateral habenula. Functional annotations delineate GluD1’s synaptic roles, encompassing the regulation of excitatory and inhibitory transmission, modulation of spine development, autophagy, and mGlu5 signaling. Clinical associations encompass behavioral flexibility, anxiety, chronic pain, depression, and schizophrenia, based on region-specific phenotypes observed in GluD1 loss-of-function models or human GRID1 mutations. Blue-outlined boxes denote brain regions where GluD1 predominantly contributes to inhibitory synaptic regulation; red boxes indicate regions where GluD1 modulates excitatory synaptic transmission or plasticity. Image panels modified from Konno et al., J. Neurosci. 2014. Abbreviations: OB olfactory bulb, MO medulla oblongata, Mb midbrain, Th thalamus, Cb cerebellum, DG dentate gyrus, LV lateral ventricle, LS lateral septum, AC anterior commissure, CeA central amygdala, LHb lateral habenula, NAc nucleus accumbens.

Taken together, GluD1 expression is not only spatially diverse but also functionally nuanced, underscoring the need to reevaluate its functional boundaries (Fig. 1). Its region- and input-specific localization provides the anatomical basis for how its modular domains are differentially recruited to regulate synaptic plasticity and behavior.

## Progress in understanding roles of GluDs based on the structural elements

GluDs share structural similarities with other iGluRs, including ATD, LBD, TMD, and CTD. Recently, there has been a surge in interest in exploring the structural relationships and functions of GluD1, leading to an expanding body of existing knowledge about them. Specific subsections are organized to discuss the structural regions relevant to GluD1.

### Amino-terminal domain

The ATD is the largest subdomain of iGluRs, comprising about 400 amino acids or one-third of the total receptor length. In GluDs, this domain facilitates tetrameric assembly through inter-subunit interactions, receptor trafficking, and trans-synaptic complex formation with cerebellin (Cblns) and presynaptic neurexin (Nxns) [42–44]. Nxns are presynaptic adhesion molecules encoded by Nrxn1-3 genes in mice and NRXN1-3 in humans, produced as longer α or shorter β forms [45, 46]. For trans-synaptic complex formation, Nxns must include alternative splice site 4 (+SS4) to interact with Cblns [43, 44, 47]. Cblns 1–4 are part of the complement component 1q (C1q)-tumor necrosis factor superfamily and show distinct expression patterns across brain regions [48–52]. Structural studies initially showed that two Nxn molecules bind to a Cbln hexamer, and two Cbln hexamers associate with one GluD, suggesting a 4:2:1 stoichiometry (4 Nxn monomers: 2 Cbln hexamers: 1 GluD tetramer) [53]. However, recent calorimetry and single-particle electron microscopy data shows each Nxns molecule binds to one Cbln hexamer. This results in a 2:2:1 stoichiometry, where two neurexins and two Cbln hexamers interact with a GluD complex, creating a physical link across the synaptic cleft to regulate synapse formation, maintenance, and plasticity [49, 54]. The reason for the apparent difference in the stoichiometry results is not readily evident since both Lee et al. and Cheng et al. used ITC with similar variants of Nrxn but obtained different results. It is likely that additional experimental details such as salts and temperature may underline the observed difference. However, single-particle electron microscopy confirms a 2:2:1 stoichiometry [54]. Importantly, this GluD1–Cbln1–Nrxn complex has also been implicated in pathological synaptic remodeling, particularly in chronic pain states, where its disruption contributes to circuit-level plasticity in the central amygdala [13, 22].

The clinical importance of these interactions is underscored by recent human genetic studies identifying several GRID1 missense mutations within the GluD1 ATD (residues 21–421), including Q117E, P120Q, R148C, P146L, R161C, K199R, A236S, R300L, and R341Q. These variants, many absent from the general population (gnomAD), are predicted to impair binding to Cbln1/2 and disrupt trans-synaptic signaling. In particular, the R341Q mutation significantly reduces GluD1–Cbln2 interaction, directly implicating ATD dysfunction in disease phenotypes [55]. These findings reinforce the critical role of this domain in maintaining synaptic integrity and raise the possibility that disruption of extracellular interactions contributes to neurodevelopmental pathology.

In addition to its role in dimer formation, the ATD of GluD receptors is essential for coupling the ATD to the LBD, which indirectly influences receptor gating [54]. Disruption of this coupling, such as by inserting a glycan wedge into the ATD-LBD linker impairs receptor function without necessarily invoking conventional ion channel activity [56]. In the A654T Lurcher mutant of GluD2 (GluD2^LC^), such disruption diminished the receptor’s sensitivity to D-serine, requiring ~1.5-fold more D-serine to inhibit the constitutive current compared to the unmodified mutant, underscoring the importance of ATD-LBD communication in maintaining ligand responsiveness [54].

Furthermore, structural analyses show that GluD ATDs have the conserved clamshell-like configuration found in iGluRs, with two lobes, R1 and R2. Unlike AMPA and kainate receptors, GluDs lack domain-swapping, where dimer partners at ATD and LBD layers cross between distal and proximal subunits [56]. Instead, GluDs show a non-swapped, linear arrangement, with the same subunit pairs forming dimers at both ATD and LBD layers, contributing to their distinct organization. Although both GluD1 and GluD2 adopt this non-swapped architecture, GluD2 displays asymmetry in its extracellular domains. One dimer arm (AB) resembles the arrangement in GluD1, while the other arm (CD) adopts a bent conformation. In this bent arm, symmetry is preserved at the ATD level but disrupted at the LBD layer, resulting in ATD arms lying in different planes. This contrasts with the upright Y-shaped architecture in AMPA and kainate receptors [54, 56, 58]. However, caution is warranted in interpreting these static structures as definitive representations of receptor conformation in vivo. Cryo-EM and crystallographic studies capture only specific states, which may not fully account for conformational flexibility or dynamic domain rearrangements during synaptic signaling. Nevertheless, this unique structure of GluDs raises questions about the evolutionary rationale behind this divergence and its functional significance. Whether these structural differences influence receptor activity, synaptic architecture, or interactions with binding partners remains an exciting area for future research.

### Ligand binding domain

Advancements in understanding GluD LBDs have revealed a structurally conserved yet functionally atypical domain. Crystallographic studies showed that the LBD of GluD2 closely resembles the GluN1 subunit of NMDA receptors [2]. Despite this structural homology, early work showed small amino acids like D-serine and glycine could bind GluD2 without triggering ion channel activation. D-serine inhibited spontaneous leak currents in GluD2^LC^, indicating ligand binding could induce conformational changes [2]. However, ligand affinity remained low, due to a unique hinge region in the LBD that limits domain closure [3]. Structural and thermodynamic analyses revealed distinct properties of GluD1. Cryo-EM and crystallographic studies showed GluDs adopts a non-swapped dimeric configuration at the ATD–LBD interface, stabilized by calcium ions and distinct from AMPA and NMDA receptors. Molecular dynamics simulations suggested a reduced LBD flexibility in GluD1, attributed to Pro725, which may restrict interlobe closure, thereby limiting channel opening transitions [59]. Thermodynamic profiling shows D-serine binds to GluD1 with higher affinity (*K*_d_ ~160 μM) than to GluD2 (*K*_d_ ~809 μM), with GluD1 binding being enthalpy-driven and exothermic, and GluD2 binding being entropy-driven and endothermic, indicating divergent recognition mechanisms [59].

The LBD of GluD1 plays a critical role in synapse formation by linking extracellular ligand recognition to intracellular signaling and trans-synaptic stability. A key residue within this domain, arginine 526 (R526), facilitates D-serine-induced conformational changes that are essential for GluD1’s non-ionotropic signaling [23]. Although R526 is conserved among iGluRs and contributes to ligand binding in GluN1, in GluD1 it appears uniquely important for synapse development [54]. Substituting R526 with lysine (R526K) disrupts the formation of inhibitory synapses onto cortical pyramidal neurons, despite normal receptor surface expression and preserved ability to form trans-synaptic complexes [39]. It was recently demonstrated that D-serine binding to the LBD destabilizes the Cbln1–GluD1 interaction at the ATD in the absence of neurexin scaffold, and this disruption is abolished in the R526K mutant [23]. Thus, R526 serves as a molecular switch coupling ligand-induced conformational signaling in the LBD with trans-synaptic reorganization at the ATD, ultimately regulating inhibitory synapse formation.

Further insights into GluD1’s molecular specificity emerged when GABA was found to bind directly to its LBD [40], a feature previously unknown among iGluRs. This binding does not trigger ion flux through the receptor but initiates a non-ionotropic signaling cascade that enhances inhibitory synaptic transmission. Mutations at key sites (R526K, and D742A) abolished the GABA-induced increase in currents at a constitutively open GluD1 mutant without affecting surface localization. The affinity of GABA for GluD1 is in millimolar range, as opposed to micromolar affinity of D-serine, suggesting that GABA most likely engages GluD1 only during high interneuron activity. Furthermore, GABA does not affect the currents at GluD2^Lc^ suggesting that it may not bind GluD2 or do so with lower affinity. These studies show the GluD1 LBD as structurally competent, ligand-sensitive, and distinct from GluD2, capable of mediating conformational signaling for synaptic modulation, even if canonical ionotropic function remains elusive.

### Ion channel pore

While structural and functional insights have established GluD1 as a ligand-sensitive receptor, whether it can act as a bona fide ion channel remains elusive. The question is not whether GluD1 contains a structurally competent ion pore; it does, but whether that pore is endogenously gated under physiological conditions.

GluDs share the conserved transmembrane topology and pore-forming domains of other iGluRs, including the M3 segment that forms the channel gate [60]. However, when expressed in heterologous systems like HEK293T cells or Xenopus oocytes, wild-type GluD1 fails to generate ligand-induced ion currents, even with known ligands like D-serine, glycine, or GABA [61]. Although constitutively active mutations like the GluD2^LC^ have been shown to unmask latent channel activity [2], notably, the same mutation in GluD1 (GluD1A654T) generates smaller leak currents surprisingly only in mouse but not rat GluD1 [59, 61, 62], highlighting functional differences between subtypes. Replacing GluD2’s LBD with those from AMPA or kainate receptors shows its transmembrane domain supports robust conductance when paired with gating-competent extracellular regions [60, 63]. Similar scenario is observed in GluD1 [36]. This implies that the gating is constrained by domain dynamics upstream, particularly at LBD and ATD levels. Studies have shown that GluD ATDs exhibit substantial conformational flexibility, which may hinder dimer stability required to couple ligand binding to pore opening [49, 57]. A shift in this perspective emerged when structurally constrained conditions revealed latent channel activity in GluDs. Carrillo and colleagues showed that GluD2’s dimer interface could be stabilized through trans-synaptic complex formation via co-expression of Cbln1 and Nrx1β or through chemical crosslinking. Under these conditions, application of glycine or D-serine evoked inward currents in HEK293T cells and rat cerebellar slices, revealing a latent inotropic function normally not found under basal conditions [64]. However, Itoh et al. challenged this interpretation by systematically examining GluD1 and GluD2 under various conditions. Using heterologous systems, they tested wild-type receptors, active mutation (GluD1^LC^ and GluD2^LC^), and an ATD-deleted GluD2 chimeric construct with D-serine, glycine, glutamate, and GABA. They also co-expressed Cblns and Nrxns to replicate trans-synaptic assembly. No ligand-induced ion currents were observed. They further showed that ligand-induced currents were not specific to GluD expression: *L*-glutamate and glycine elicited similar responses in naive HEK293 cells, GluD2-null Purkinje neurons, and controls. These results suggested that the observed currents might arise from artifacts or non-GluD-related activity or specific experimental conditions which are harder to identify [61]. This indicates that GluDs, although architecturally ionotropic, lack intrinsic gating under standard conditions and operate as scaffold-like or modulatory receptors.

The absence of channel activity in vitro contrasts with in vivo evidence suggesting GluD1 can mediate ion conductance under specific physiological conditions. In Purkinje cells, activation of Gq-coupled mGluR1 receptors initiates GluD2 pore opening via downstream pathways [65, 66]. Similarly, for GluD1, studies show that metabotropic mechanisms can induce its channel activity. In midbrain dopamine neurons, activation of mGluR1/5 receptors leads to GluD1 channel opening. This was shown by the induction of slow excitatory postsynaptic currents (sEPSCs) upon applying mGluR1/5 agonist DHPG in neurons co-expressing GluD1 and mGluR1/5. These currents were abolished by mGluR1/5 antagonists and by expressing a dominant-negative GluD1 mutant, confirming mGluR1/5’s role in gating GluD1 channels [67]. In the dorsal raphe nucleus, GluD1 contributes to tonic excitatory conductance modulating neuronal resting potential [68]. Similar findings in the BNST show GluD1 supports a persistent tonic inward current that is independent of classical glutamatergic transmission. Conley et al. recently identified a non-synaptic, GluD1-dependent current in BNST neurons that was abolished following GluD1 knockdown [69]. This tonic current, which enhances neuronal excitability, occurred independently of discrete synaptic events and was not mediated by activation of classical iGluRs. These studies support a model in which GluD1 contains a latent ion channel that can be selectively engaged under specific regional contexts, possibly through distinct molecular interactions, signaling cascades, or synaptic configurations. The ion channel enigma of GluDs however still remains to be clearly resolved. Interestingly, the invertebrate GluD is readily activated by GABA [70] suggesting that there are evolutionary differences which may need to be considered to solve this mystery.

### C-terminal domain

GluD1 contains a C-terminal PDZ-binding motif, similar to other iGluRs, but interacts weakly with classical scaffold proteins like PSD-95 [63]. Deleting this PDZ-binding sequence minimally affects synaptic localization. However, removing the entire CTD disrupts synaptic targeting, despite normal surface expression. This shows GluD1 trafficking depends not on the canonical PDZ motif but on distinct noncanonical elements within the CTD for synaptic localization [63, 71, 72]. Other proteins, including Shank, PICK1, PSD93, and EMAP, also interact with the CTD of GluD1 and/or GluD2 [73–76]. The contribution of these CTD elements has been further examined using chimeric and domain-swap experiments that reveal the molecular logic of GluD1 trafficking. Replacing GluD1’s LBD with that of GluK2 restored synaptic localization but did not rescue LTP, suggesting that receptor trafficking to the synapse and the ability to support synaptic plasticity are governed by distinct mechanisms. Additionally, substituting GluD1’s PDZ-binding motif with that of TARPγ-8 restored receptor function but failed to promote synaptic delivery, indicating that GluD1 trafficking is regulated by specific sequence elements beyond functional gating. Deletion mapping identified a 10-amino-acid sequence “QNTQLSVSTF” in the proximal CTD as essential for synaptic localization. Within this sequence, threonine at position 923 (T923) emerged as a key regulatory residue. A single-point mutation substituting T923 with alanine (T923A) disrupted receptor trafficking, while replacing it with aspartate (T923D) restored proper localization, suggesting that phosphorylation at T923 mediates interactions with trafficking partners. However, T923 was not phosphorylated by common kinases such as PKA, PKC, or CaMKII, suggesting the involvement of a noncanonical kinase or a phosphorylation-independent mechanism [63]. In contrast, a nearby serine residue in GluD2 (S945) is essential for PKC-dependent LTD at cerebellar PF-PC synapses. S945 phosphorylation by PKC is required for receptor internalization and plasticity following LTD-inducing stimulation [77, 78]. However, subsequent studies have challenged this model, showing that LTD can still occur in the absence of S945 phosphorylation, indicating that this residue may not be universally required for plasticity [79]. These contrasting findings suggest that while T923 in GluD1 and S945 in GluD2 occupy homologous positions, their roles diverge in terms of kinase sensitivity and functional outcomes. T923 regulates trafficking independently of common kinases, whereas S945 mediates PKC-dependent LTD, highlighting subtype-specific regulatory mechanisms.

Evidence supporting the idea that the GluD1 CTD acts as a transsynaptic signaling hub comes from studies demonstrating its involvement in organizing postsynaptic scaffolds and interacting with presynaptic partners. Remarkably, even short CTD motifs of 5-13 amino acids can relay presynaptic identity into selective postsynaptic responses. Nrxn1 with Cbln2 enhances NMDA receptor-mediated transmission, while Nrxn3 with Cbln2 suppresses AMPA receptor activity, without requiring ion conduction or changes in synapse number. Minimal GluD1 constructs with only the Cbln-binding ATD, an unrelated transmembrane domain, and a relevant CTD motif reproduced these bidirectional outcomes [37]. Notably, the motif for NMDA enhancement includes a serine residue homologous to GluD2 S945, supporting a shared mechanism by which GluDs translate extracellular signals into receptor-specific plasticity [78]. Together, these findings reframe GluD1 CTD as using distinct motifs to orchestrate receptor trafficking and plasticity, potentially through a phosphorylation mechanism to relay transsynaptic signals.

## Targeting GluD1 domains: novel interventions for synaptic dysfunction and neurodevelopmental disease

Although the contributions of GluD1 domains to synaptic and circuit function were discussed in earlier sections, it is equally crucial to note how this domain-level understanding is currently guiding therapeutic approaches for disease intervention and circuit repair.

### Extracellular scaffolding and ATD-targeted interventions

**Fig. 2:**
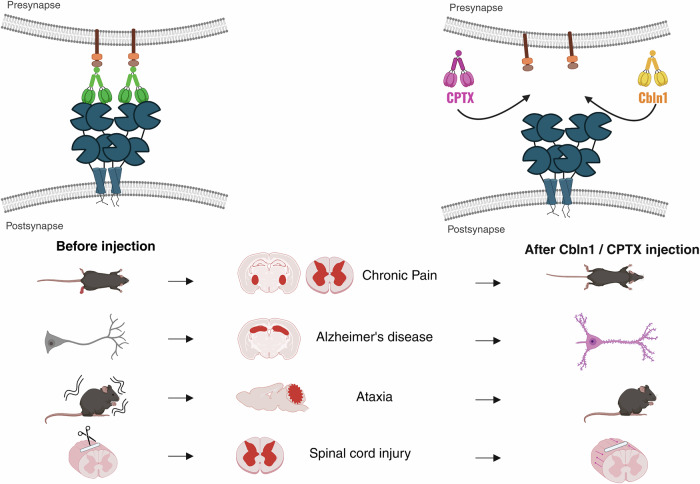
Synapse-restoring potential of Cbln1 and synthetic synaptic organizer CPTX across neurological disease models. The top panels depict the molecular mechanism underlying synaptic restoration. On the left, under pathological conditions such as chronic pain, Alzheimer’s disease, ataxia, and spinal cord injury, there in synaptic destabilization. On the right, the exogenous administration of Cbln1 or CPTX reestablishes transsynaptic signaling by bridging presynaptic neurexins and postsynaptic GluD1 or AMPA receptors, thereby restoring synaptic integrity. The bottom panels illustrate the therapeutic outcomes observed across various disease models. Before the injection of Cbln1/CPTX, animals exhibit behavioral and structural deficits associated with chronic pain, neurodegeneration, motor dysfunction, and neural injury. Following the injection, synaptic restoration is correlated with improved neuronal structure, behavioral recovery, and functional circuit repair. These findings underscore the translational potential of targeting extracellular scaffolds such as Cbln1 and synthetic organizers like CPTX for the restoration of circuit function in a range of neurological disorders.

Our recent work demonstrates the importance of GluD1 in pain-associated plasticity within the central amygdala and spinal cord [13, 22, 25]. In rodent models of chronic inflammatory and neuropathic pain, downregulation of GluD1 and Cbln1 in the latero-capsular division of the central amygdala (CeLC) disrupts excitatory synaptic homeostasis in a time-dependent and lateralization manner, leading to increased AMPA receptor expression and hyperexcitability. These changes correlate with enhanced nocifensive behavior. Remarkably, systemic administration of recombinant Cbln1 restores GluD1-dependent signaling, normalizes AMPAR levels, and alleviates pain phenotypes, an effect absent in GluD1 KO (Fig. 2). The specificity of this intervention is further highlighted by the ineffectiveness of the Cbln1 mutant (Cbln1^Y122A/R124A/D147A^) that cannot bind to GluD1 as well as the observation that D-serine can disrupt GluD1–Cbln1 binding and mimic the effects of GluD1 KO, suggesting that ligand-sensitive modulation of this interaction is a viable therapeutic avenue [13, 22]. Echoing this concept the synthetic synaptic organizers such as CPTX, which bridges neurexin and AMPA receptors, have been demonstrated to restore excitatory synapses and improve motor coordination in GluD2- and Cbln1-deficient mice, rescue memory and synaptic function in 5XFAD Alzheimer’s models, and even reorganize excitatory circuits to restore locomotion following spinal cord injury [80] (Fig. 2). Although CPTX does not directly engage GluD1, its mechanism of action parallels the transsynaptic scaffolding functions of the GluD1–Cbln1 complex and offers a promising blueprint for developing ATD-targeted therapeutics.

### Ligand recognition and LBD-targeted modulation

While the ATD mediates trans-synaptic anchoring, the LBD of GluD1 functions as a conformational switch that translates extracellular signals into intracellular responses independently of ion flux. Structural studies on the GluD2^LC^ revealed that orthosteric ligands such as 7-chlorokynurenic acid and halogenated alanine analogs induce partial cleft closure of the LBD, stabilizing intermediate conformations [3]. These ligand-induced changes modulate downstream signaling without opening the ion channel, highlighting the therapeutic potential of targeting receptor conformation over conventional gating mechanisms. These findings offer a structural rationale for designing GluD1-selective, non-ionotropic modulators. Recent work extends this framework to GluD1. Its LBD binds GABA, enhancing IPSCs via a conformational, non-ionotropic mechanism that promotes long-term inhibitory plasticity [40]. In contrast, D-serine binding disrupts the GluD1–Cbln1 interaction, leading to the breakdown of trans-synaptic scaffolds in pain-related circuits and mimicking GluD1 knockout phenotypes, such as AMPA receptor upregulation in the central amygdala [23].

**Fig. 3:**
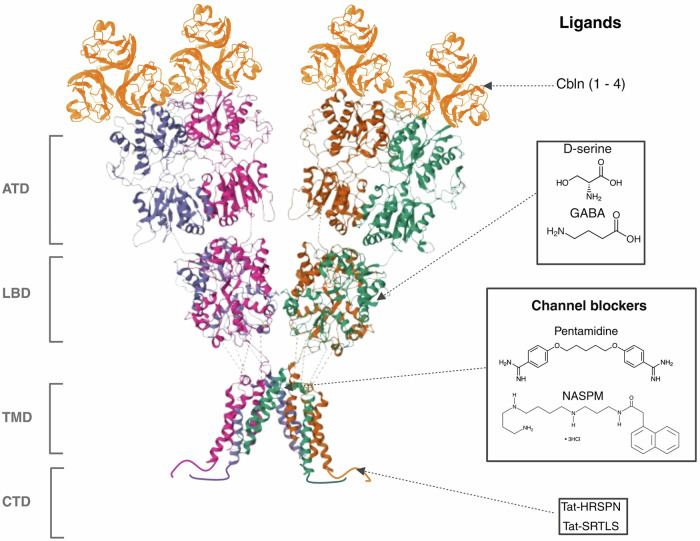
Domain architecture of GluD1 and current therapeutic strategies targeting its modular structure. The structural model of the GluD1 receptor delineates its four principal domains: the amino-terminal domain (ATD), ligand-binding domain (LBD), transmembrane domain (TMD), and carboxy-terminal domain (CTD). Each domain is associated with emerging pharmacological interventions or interacting ligands. At the extracellular ATD, cerebellin family proteins (Cbln1–4) bind to form transsynaptic scaffolds that are crucial for synapse formation and maintenance. Within the LBD, GluD1 interacts with ligands such as D-serine and GABA, which induce conformational changes that modulate synaptic plasticity through non-ionotropic mechanisms. The TMD, although structurally capable of forming a channel, exhibits latent ionotropic activity; gain-of-function mutants or engineered conditions reveal channel activity that can be inhibited by small-molecule blockers such as pentamidine and NASPM. The intracellular CTD contains essential motifs for trafficking and signaling; short synthetic peptides (e.g., Tat-HRSPN and Tat-SRTLS) derived from this region have demonstrated therapeutic efficacy in restoring synaptic function and reversing circuit-level plasticity in chronic pain models. This figure illustrates the therapeutic significance of targeting GluD1’s domain-specific interactions and downstream signaling cascades in neurological and neuropsychiatric disorders.

Together, these findings establish the GluD1 LBD as a ligand-sensitive, bidirectional modulator of synaptic architecture (Fig. 3). Targeting this domain with stabilizers or disruptors offers a promising strategy for correcting circuit-level dysfunction in conditions such as chronic pain, stress disorders, and neurodevelopmental diseases.

### Ionotropic modulation: therapeutic potential of channel blockade

Despite the ongoing debate on the ion channel activity of GluDs, its gain-of-function mutation (GluD1–A650T and GluD2^LC^) which generate leak currents, can be blocked by small molecules, including some FDA-approved drugs. For instance, pentamidine effectively suppresses currents via constitutively open variants GluD2-T649A and GluD1-A650T at nanomolar and micromolar concentrations respectively. Similarly, memantine, a drug used to block NMDA receptors in clinical settings, partially decreases leak currents in GluD1/2 Lurcher variants [55]. While current IC_50_ values may limit immediate clinical translation, these findings underscore the feasibility of targeting GluD leak conductance pharmacologically (Fig. 3). Importantly, such channel blockers may hold particular therapeutic potential in brain regions where tonic GluD-mediated currents are functionally relevant, such as dopaminergic neurons, the dorsal raphe nucleus, and BNST, highlighting a potential for region-specific precision interventions in neuropsychiatric conditions.

### Intracellular signaling and CTD-directed therapies

**Fig. 4:**
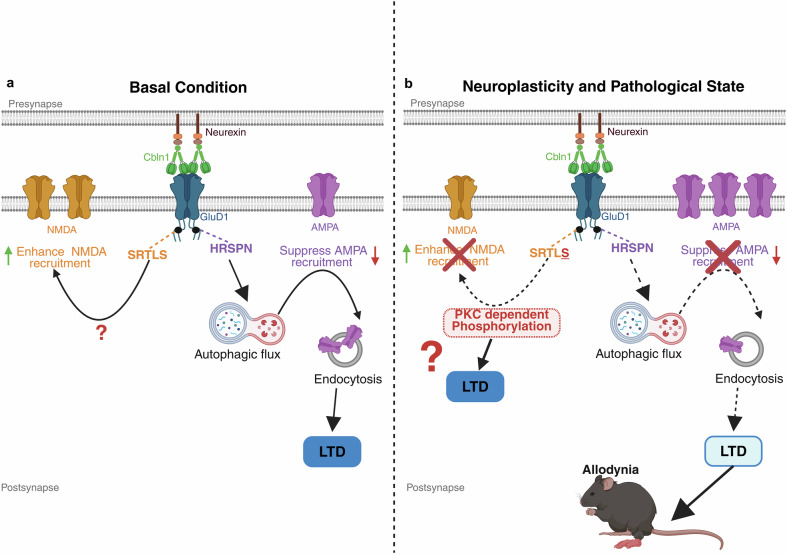
C-terminal domain-specific signaling by GluD1 in basal and pathological states: Mechanistic insights and peptide-based rescue strategies. **a** Under basal conditions, GluD1 integrates transsynaptic input from neurexin–Cbln1 complexes to regulate NMDA and AMPA receptor trafficking and autophagic signaling. The GluD1-region SRTLS enhances NMDA receptor recruitment, while HRSPN promotes autophagic flux and suppresses AMPAR surface expression, both converging on mechanisms that promote long-term depression (LTD) and synaptic remodeling. **b** In neuroplastic or pathological states such as chronic pain, these signaling dynamics are disrupted. One hypothesis is that PKC-dependent phosphorylation impairs NMDA receptor recruitment, and excessive AMPAR surface accumulation leads to circuit hyperexcitability and pain hypersensitivity (allodynia). Administration of HRSPN peptide can partially restore autophagy and normalize receptor trafficking, rescuing LTD mechanisms and mitigating pathological outcomes. This figure highlights the modular functionality of GluD1 and its CTD motifs as intracellular effectors linking transsynaptic cues to synaptic plasticity, with translational potential in pain and neuropsychiatric conditions.

Beyond extracellular scaffolding, domain-specific interventions also extend to intracellular pathways governed by the CTD. Conditional deletion of GluD1 from corticolimbic excitatory neurons leads to hyperactivation of the Akt–mTOR pathway, a known suppressor of autophagy, resulting in elevated p62 levels and reduced LC3-II/LC3-I ratios across key forebrain regions [11]. In pain states, these autophagic impairments were particularly evident in GluD1-expressing PKCδ+ neurons of the CeLC. A synthetic peptide (Tat-HRSPN) mimicking the distal CTD of GluD1 (GluD1^969-973)^ remarkably (Fig. 3), restored autophagic signaling, reduced AMPAR-mediated mEPSCs, and reversed mechanical hypersensitivity even in GluD1 KO animals (Fig. 4) [25]. Together, these findings establish the CTD as an autonomous signaling hub capable of rescuing circuit plasticity even in the absence of full-length receptor expression.

Emerging genetic evidence further supports CTD’s relevance to neurodevelopment. Homozygous GRID1 mutations have been identified in individuals with intellectual disability and spastic paraplegia. Functional studies reveal that these variants disrupt mGlu1/5 receptor signaling, impair synaptic maturation, and reduce excitatory synapse density, phenotypes consistent with CTD-linked effector dysfunction [81]. Together, these findings position the GluD1 CTD as a critical integrator of intracellular signaling, with therapeutic potential for restoring circuit function in both disease and developmental contexts.

## Difference between GluD1 and GluD2

Although in this review we have used GluD2 information where GluD1 knowledge is lacking, it should be noted that there are both similarities and major differences in GluD1 and GluD2. At the level of synaptogenic activity, the role of GluD1 at inhibitory synapses is well understood as is the affinity of GABA for GluD1 LBD. There are major structural differences of full length GluDs (lacking the Cbln-Nrxn scaffold) with GluD1 having a more symmetric ATD-LBD conformation compared to GluD2. While lurcher mutation produces robust constitutive activity in GluD2 but not in GluD1. Similarly tonic activity in native system has been more often tested with GluD1. With respect to disease relevance the roles seem to follow preferential expression, with GluD2 mainly related to ataxia and GluD1 relevant to social behaviors, chronic pain, striatal motor function and drugs of abuse-induced plasticity. It remains to be seen whether these differences are due to lack of full understanding of the ion channel activity or cases where studies have been focused on one or the other subunit.

## Conclusion

Once regarded as a functionally ambiguous receptor, GluD1 is now recognized as a structurally modular and functionally versatile regulator of synaptic organization, neurodevelopmental processes, and circuit-level plasticity. Rather than mediating classical ionotropic signaling, GluD1 engages in context-dependent mechanisms ranging from extracellular interactions with Cblns and Nrxn to intracellular modulation of autophagy and synaptic receptor composition. These diverse roles are governed by distinct structural domains, whose dysfunction has been implicated in a range of pathological states, including chronic pain and neuropsychiatric disorders. The development of domain-specific therapeutic interventions, such as peptide-based modulators and synthetic synaptic organizers, underscores the translational relevance of dissecting GluD1’s molecular architecture. However, several key questions remain regarding its endogenous activation mechanisms, domain crosstalk, and contributions to disease etiology. Continued investigation into these unresolved aspects will be essential for advancing GluD1 as a promising therapeutic target in brain disorders.

## Outstanding Questions

1. ***What structural principles govern GluD1’s synaptic targeting across excitatory and inhibitory synapses?*** GluD1 selectively localizes to unique synaptic subtypes and compartments, yet the molecular logic behind this specificity, particularly the role of CTD motifs or ATD–LBD interactions, remains elusive.
2. ***What molecular or cellular conditions enable endogenous gating of the GluD1 ion channel in vivo?*** Despite structural evidence of a functional pore, GluD1 remains inactive under recombinant conditions. Identifying physiological contexts, co-factors, or signaling cascades that unlock this latent conductance remains a critical challenge.
3. ***How do ligand-specific and non-ionotropic signaling mechanisms coordinate across GluD1 domains?*** Ligand binding to the LBD initiates conformational changes without ion flow. Whether this signaling is mechanically linked to CTD motifs or modulated through domain-specific crosstalk is unknown.
4. ***How does GluD1 integrate transsynaptic signals to direct postsynaptic remodeling and plasticity?*** The discovery of short CTD motifs that differentially modulate AMPA and NMDA receptor activity suggests that GluD1 acts as a molecular relay. But how these motifs are regulated, and whether their function is plastic or state-dependent, is an open question.
5. ***Can GluD1 dysfunction be causally linked to neurodevelopmental and psychiatric disorders?*** Genome-wide association and patient-based studies implicate GRID1 mutations in schizophrenia and intellectual disability. Future research should explore how these variants alter GluD1’s developmental, synaptic, and circuit-level functions.
6. ***What is the therapeutic potential of modulating GluD1-Cbln signaling in chronic pain and neuropsychiatric disorders?*** Recent findings point to Cbln1 as a modifiable partner in GluD1-related plasticity. Understanding how ligand competition, scaffold disruption, or exogenous restoration affects circuit outcomes may open new avenues for disease-specific interventions.

## GenAI disclosure statement

Generative AI tools, including ChatGPT 4.0 and Paperpal, were used exclusively to assist with grammar refinement and writing clarity in select sections of this manuscript. All scientific content, interpretations, and conclusions are entirely the authors’ original work. The authors have thoroughly reviewed and edited the text and take full responsibility for the final content of the published article.

## Data Availability

No data was used for the research described in the article.
